# Biosynthesis of Silver Nanoparticles Using *Salvia pratensis* L. Aerial Part and Root Extracts: Bioactivity, Biocompatibility, and Catalytic Potential

**DOI:** 10.3390/molecules28031387

**Published:** 2023-02-01

**Authors:** Nikola Z. Srećković, Zoran P. Nedić, Daria Maria Monti, Luigi D’Elia, Silvana B. Dimitrijević, Nevena R. Mihailović, Jelena S. Katanić Stanković, Vladimir B. Mihailović

**Affiliations:** 1 University of Kragujevac, Faculty of Science, Department of Chemistry, Radoja Domanovića 12, 34000 Kragujevac, Serbia; 2 University of Belgrade, Faculty of Physical Chemistry, Studentski Trg 12-16, 11159 Belgrade, Serbia; 3 University of Naples Federico II, Department of Chemical Sciences, Complesso Universitario Monte Sant’Angelo, via Cinthia 4, 80126 Naples, Italy; 4 eLoop S.r.l., Viale Antonio Gramsci 17/B, 80122 Napoli, Italy; 5 Mining and Metallurgy Institute Bor, Zeleni Bulevar 35, 19210 Bor, Serbia; 6 University of Kragujevac, Institute for Information Technologies Kragujevac, Department of Science, Jovana Cvijića bb, 34000 Kragujevac, Serbia

**Keywords:** *Salvia pratensis* L., silver nanoparticles, catalysts, hemolytic activity, antimicrobial activity, antioxidant activity

## Abstract

The aim of this research was the synthesis of silver nanoparticles (SPA- and SPR-AgNPs) using the aqueous extracts of the aerial (SPA) and the root (SPR) parts of the plant *Salvia pratensis* L., their characterization, reaction condition optimization, and evaluation of their biological and catalytic activity. UV–Vis spectroscopy, X-ray powder diffraction (XRPD), scanning electron microscopy with EDS analysis (SEM/EDS), and dynamic light scattering (DLS) analysis were utilized to characterize the nanoparticles, while Fourier transform infrared (FTIR) spectroscopy was used to detect some functional groups of compounds present in the plant extracts and nanoparticles. The phenolic and flavonoid contents, as well as the antioxidant activity of the extracts, were determined spectrophotometrically. The synthesized nanoparticles showed twice-higher activity in neutralizing 2,2′-azino-bis(3-ethylbenzothiazoline-6-sulfonic acid) (ABTS^+^) compared with the respective extracts. SPR-AgNPs exhibited strong antimicrobial activity against almost all of the tested bacteria (<0.0039 mg/mL) and fungal strains, especially against the genus *Penicillium* (<0.0391 mg/mL). Moreover, they were fully biocompatible on all the tested eukaryotic cells, while the hemolysis of erythrocytes was not observed at the highest tested concentration of 150 µg/mL. The catalytic activity of nanoparticles toward Congo Red and 4-nitrophenol was also demonstrated. The obtained results confirm the possibility of the safe application of the synthesized nanoparticles in medicine and as a catalyst in various processes.

## 1. Introduction

Due to nanoscale size, distinct geometries and higher surface area, nanoparticles have different properties than micrometer-sized particles that have essentially no activity. These properties have brought nanoparticles to the center of interest for a variety of applications, particularly in biomedicine, biotechnology, and electronics [1]. Today, the highest percentage of synthesized nanoparticles is obtained by physical and chemical methods that require the use of high pressures, temperatures, and chemicals that have a devastating impact on the environment. In addition, the use of chemicals limits the application of such nanoparticles for biomedical purposes [2]. For this reason, researchers have been working to develop new methods of synthesis that do not require the use of enormous amounts of energy and toxic chemicals. Microorganisms, such as bacteria, fungi, and algae, as well as plant extracts are currently used in extensive research on the biological synthesis of nanoparticles of various metals. While the synthesis of nanoparticles using microbes has not proven to be much efficient due to the length of the reaction, the need for previous preparation, and the risk of contamination, the synthesis of nanoparticles using plants has grown in importance. Over 200 plant species from various families have been used to examine the feasibility of producing different metal nanoparticles [3]. The main interest in nanoparticles is based on the concept that, thanks to their small size, they can interact with the cell membrane and various proteins, thus having the ability to enter hard-to-reach areas, such as the brain tissue [4]. The catalytic reduction of pollutants is an important part of pollution control. Catalytic reduction is a process that uses a catalyst to reduce the amount of pollutants released into the environment. This process is widely used to reduce the emissions of gases, dyes, and other pollutants from various sources. It has been demonstrated that metallic nanoparticles also have catalytic properties and their use in pollution control is quite popular nowadays [5]. In addition to catalytic reduction, other methods of pollutant removal include chemical oxidation, adsorption on biomass, and micellar solubilization. Chemical oxidation involves the use of an oxidizing agent to convert pollutants into less hazardous substances. Adsorption on biomass uses biological materials such as algae and bacteria to trap pollutants and prevent them from entering the atmosphere. Micellar solubilization uses surfactants to break down pollutants into smaller particles that can be easily removed from the environment. In this process, nanostructured carriers are often included. The use of nanosized particles for this purpose is considered a cost-effective and safe process that can reduce the amount of pollutants released into the environment [6,7].

Today, there is a big trend in the world to reduce the consumption of energy and resources and increase the efficiency of many processes. For this reason, the focus of this research was the synthesis of silver nanoparticles using renewable resources, i.e., plants, with less energy consumption and in a brief time. The role of primary and secondary plant metabolites is reflected in their ability to reduce silver ions and prevent the formation of large agglomerates that exceed the dimensions that describe nanoparticles. Additionally, plant metabolites enhance the solubility of synthesized nanoparticles by increasing the polarity of their surface whereby one end of the molecule is attached to the nanoparticle, and the other is oriented toward the solvent. However, the use of plants in the biosynthesis of nanoparticles results in polydispersed nanoparticles whose size ranges from 5 to 200 nm, which can significantly affect their biological activity. The most frequently synthesized nanotechnology products are silver nanoparticles (AgNPs), which have special qualities, including high chemical stability, antibacterial, antiviral, antioxidant, anti-inflammatory, anticancer activities, and conductivity. Their usage as an alternative to antibiotics, particularly in wound healing, has been extensively researched [8]. Moreover, they also exhibit minimal cytotoxicity and immunological reaction and are inexpensive. Precisely because of these properties, silver nanoparticles today have multiple medical applications: drug delivery, molecular diagnostics, medical imaging, making artificial joint replacements, dressing wounds as well as drugs to promote wound healing [9].

This is not the first study to use the sage species in the biosynthesis of nanoparticles. Since *Salvia* plants have one of the longest histories of medicinal use, many attempts have been made to improve the plant’s biological activity. The synthesis of different types of nanoparticles using these plants, in which the active polyphenolic compounds of the plants are incorporated into the nanoparticles themselves, has been reported. Metwally et al. [10] successfully synthesized silver nanoparticles using *Salvia officinalis* leaf extract, whereby the formed nanoparticles showed quite good anti-inflammatory potential. Another research confirmed the success in the synthesis of silver nanoparticles using *S. officinalis* leaf extract and the obtained nanoparticles showed catalytic activity during the degradation of the synthetic color Congo Red [11]. Other researchers showed that silver nanoparticles obtained using *S. officinalis* possessed antioxidant, antibacterial, antileishmanial, antiplasmodial, and cytotoxic activities [12,13,14]. In addition to the most famous *S. officinalis*, other varieties of sage have also been utilized to synthesize nanoparticles. Silver nanoparticles with good antioxidant activity were successfully synthesized with *S. aethiopis* [15], whereas silver nanoparticles synthesized using *S. leucantha* possessed strong antibacterial activity [16].

In our previous research, we examined the chemical composition and bioactivity of the aerial part and roots in methanolic extracts of the plant *S. pratensis*. The results showed that this unjustly neglected *Salvia* species is extremely rich in the content of various polyphenolic compounds. The root was richer in phenolic acid content, while the aerial part possessed a higher flavonoid content. Additionally, the root extract showed higher antioxidant, antimicrobial, and cytotoxic activities [17]. The synthesis of silver nanoparticles using aqueous extracts of the aerial part (SPA-AgNPs) and roots (SPR-AgNPs) of *S. pratensis* aimed to obtain biologically active and biocompatible nanoparticles (SPA- and SPR-AgNPs) with low energy consumption and without the use of harmful chemicals. Moreover, the antioxidant, antimicrobial, cytotoxic, and hemolytic activities of the nanoparticles were evaluated.

## 2. Results and Discussion

### 2.1. Optimization Conditions and Synthesis of Silver Nanoparticles

**Figure 1:**
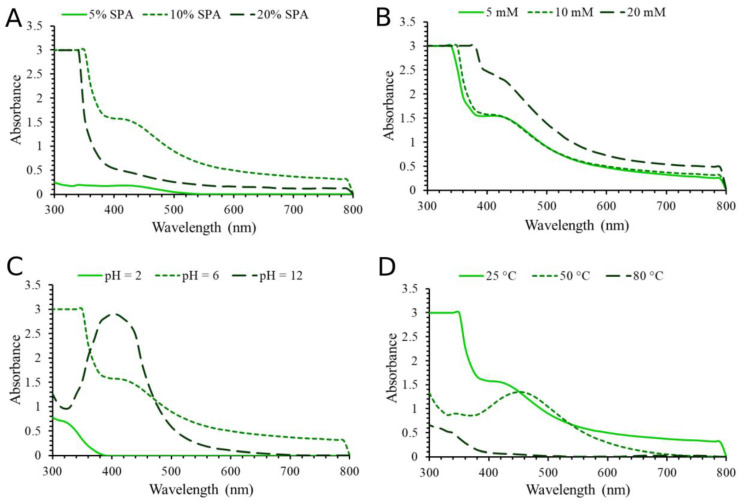
Influence of different parameters on the synthesis of SPA-AgNPs. (**A**) Extract concentrations, (**B**) AgNO_3_ concentrations, (**C**) pH value, and (**D**) temperature.

The yield, quality, and characteristics of the formatted AgNPs could be considerably influenced by the ratio of the plant extract to the metal ions, the length of the synthesis process, the temperature, and the pH of the reaction [18]. Silver nanoparticles (SPA-AgNPs and SPR-AgNPs) used for the characterization, biological, and catalytic tests were synthesized by combining the most favorable conditions obtained by the previous optimization. For the environmentally friendly synthesis, the extraction of plant material and the synthesis of nanoparticles took place in an aqueous environment. The formation of silver nanoparticles was monitored by recording the spectrum from 800 to 300 nm using a UV–Vis spectrophotometer, where the highest absorption peaks were located from 400 to 440 nm. In this range of wavelengths, there is an oscillation of electrons on the surface of the nanoparticles, which is reflected by the absorbance, and this phenomenon is called surface plasmon resonance [19]. In addition, visual color changes from light yellow to dark brown confirm the reduction of silver ions to their elemental form. Figure 1 shows the dependence of the synthesis of silver nanoparticles using the aerial part extract of the plant depending on the change in parameters such as the concentration of the extract, the concentration of AgNO_3_, temperature, and pH value. As fixed conditions, 10 mM AgNO_3_ solution, 10% extract solution, room temperature, and pH value 6 were used. By changing one parameter at a time, the change in the absorption spectrum was monitored using the spectrophotometer.

The effect of changing the concentration of the SPA on the formation of silver nanoparticles is shown in Figure 1A. It can be noted that the 10% extract solution showed the highest absorption peak indicating the highest nanoparticles yield, while the formation of nanoparticles was significantly lower using 20% and 5% concentrations. The choice of plants for the biosynthesis of nanoparticles is a very important parameter. Although most of the different plant species contain biomolecules that can act as reducing and stabilizing agents during synthesis, there are plants that are rich in the content of some compounds such as tannins, which can lead to the formation of agglomerates. The surface characteristics of AgNPs may also be influenced by these phytomolecules [20,21]. An increase in the concentration of silver nitrate (Figure 1B) had a favorable effect on the formation of nanoparticles. The increase in pH value (Figure 1C) also promoted the formation of silver nanoparticles. It was observed that by increasing the pH value, the absorption maximum shifted to lower wavelengths. This phenomenon can be explained by the fact that the optical properties of spherical silver nanoparticles depend on their diameter. Larger nanoparticles show higher scattering and have broadened and shifted peaks toward longer wavelengths, whereas smaller nanoparticles predominantly absorb light and have peaks around 400 nm [22]. Interestingly, an increase in temperature from 25 to 50 °C (Figure 1D) had a positive effect on the synthesis, while a further increase had a negative effect, shown as an agglomeration of NPs that was visually noticeable.

**Figure 2:**
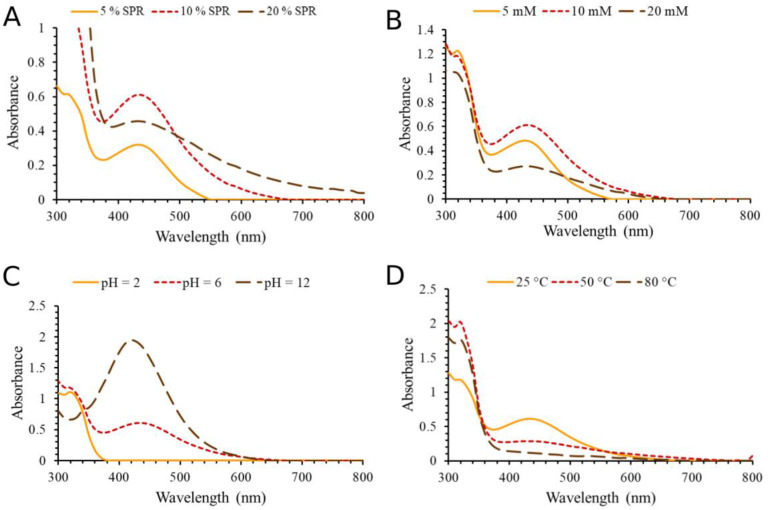
Influences of different parameters on the synthesis of SPR-AgNPs. (**A**) Extract concentrations, (**B**) AgNO_3_ concentrations, (**C**) pH value, and (**D**) temperature.

Likewise, a 10% root extract solution showed a higher AgNPs yield (Figure 2A). In this case, AgNO_3_ at a concentration of 10 mM was the most effective in formation of AgNPs (Figure 2B), while the test with 20 mM concentration caused the visible formation of AgNPs agglomerates and lowest AgNPs yield. Like the synthesis of nanoparticles with SPA, increasing the pH value had a beneficial effect (Figure 2C). The increase in temperature (Figure 2D) in synthesis AgNPs with SPR had a negative effect on the formation of nanoparticles that is significant in terms of environmental protection (i.e., energy efficiency). The main objective of the green synthesis of AgNPs is to increase safety and dependability while minimizing the environmental and financial harm caused by hazardous raw materials.

**Figure 3:**
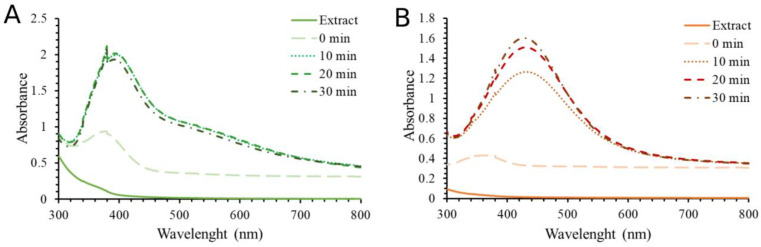
Time-dependent UV–Vis absorption spectra of (**A**) SPA-AgNPs and (**B**) SPR-AgNPs obtained under the optimal conditions.

After the optimization was completed, both types of silver nanoparticles were synthesized by combining the most favorable conditions (Figure 3A,B). The most optimal conditions for SPA-AgNPs synthesis were 10% SPA; 20 mM AgNO_3_; pH = 6; t = 50 °C, while for SPR-AgNPs synthesis, they were 10% SPA; 10 mM AgNO_3_; pH = 6; t = 25 °C. The reaction took place until the highest absorption maximum was reached (30 min) and the nanoparticle solutions were centrifuged at 12,000 rpm for 20 min. After deposition, the nanoparticles were washed with deionized water, dried at room temperature, and stored in a cool and dark place.

### 2.2. Characterization of the Obtained AgNPs

#### 2.2.1. XRPD Analysis

**Figure 4:**
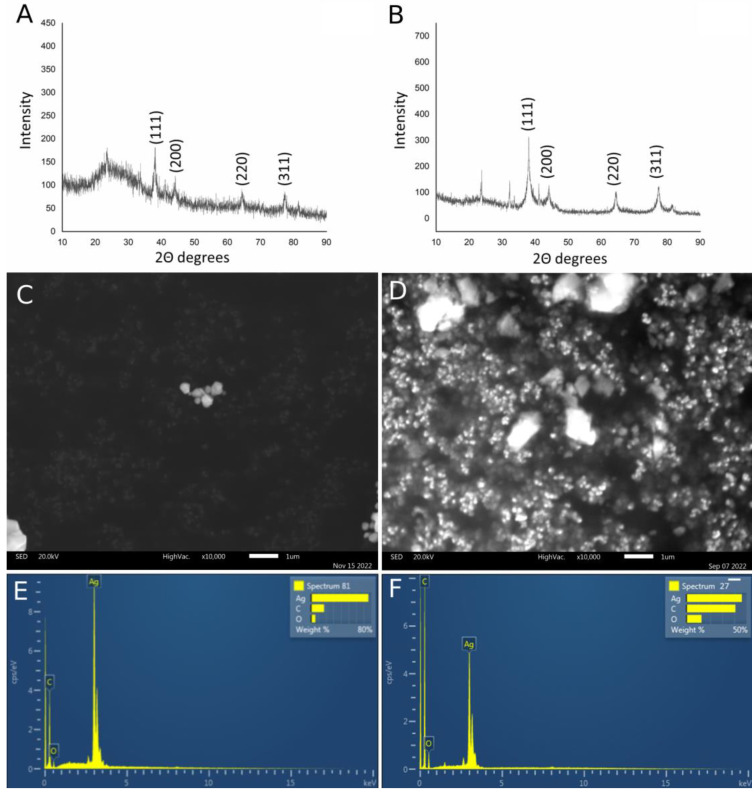
Characterization of AgNPs synthesized using *S. pratensis* extracts using various analytical techniques. XRPD spectrum of (**A**) SPA-AgNPs and (**B**) SPR-AgNPs; SEM images of (**C**) SPA-AgNPs and (**D**) SPR-AgNPs and their corresponding EDS spectra ((**E**) and (**F**), respectively).

In order to confirm the crystal structure of the synthesized silver nanoparticles, the nanoparticles were subjected to X-ray structural analysis by XRPD. The obtained results for SPA-AgNPs and SPR-AgNPs are shown in Figure 4A,B, respectively. It was determined that there were crystalline phases present based on the measured I/Imax intensities, reciprocal intervals, comparability to the literature data, and ICDD standard (PDF 87-0597) [2].

Through a comparison with the standard, the obtained XRPD spectrum confirmed the crystalline nature of the synthesized nanoparticles, indicating their nanocrystalline form. Silver nanoparticles synthesized by the aerial part of the aqueous extract of *S. pratensis* exhibited Bragg diffraction peaks at 38.14°, 44.22°, 64.4°, and 77.34°, respectively, in the X-ray powder diffractograms. The peaks corresponded to the face-centered cubic (fcc) crystalline silver planes (111), (200), (220), and (311), respectively. A slightly better spectrum, with much less noise, was obtained by recording nanoparticles synthesized using the root extract with peaks at 38.04°, 44.16°, 64.54°, and 77.5°.

The presence of some bioorganic chemicals in the extracts that can be attached to the surface of the silver nanoparticles such as proteins, carbohydrates, and phenolic compounds may correspond to a few unidentified peaks [23,24]. There have been reports of similar results when silver nanoparticles were synthesized using extracts of other sage species [11,15], *Lythrum salicaria* [2], horse chestnut [24], pulp mill wastes [25], and many other plant materials.

#### 2.2.2. SEM/EDS Analysis

In order to describe the morphology of the obtained nanoparticles, the SEM technique was used. Through this analysis, it was confirmed that both types of obtained nanoparticles were spherical in shape, while the larger agglomerates had a slightly hexagonal structure (Figure 4C,D). The appearance of agglomerates occurred as a result of sample preparation for this type of analysis, which requires their drying, and as a result, their aggregation occurs. However, a large number of small particles of nano dimensions were also visible. Their nano-dimension was confirmed by DLS analysis, which was recorded from a solution of nanoparticles, these results will be discussed in the next section. EDS analysis was used to detect the elements found in the nanoparticle structure. Based on the obtained spectra, both types of nanoparticles consisted of silver (in the highest percentage), followed by carbon and oxygen. Identified oxygen in the EDS spectra of synthetized AgNPs most likely originated from polyphenolic compounds. AgNPs synthesized using SPR contained a higher percentage of carbon and oxygen than SPA-AgNPs, suggesting that SPR-AgNPs may contain a higher amount of phytocompounds, primarily phenolic compounds, bound to the nanoparticles’ surface.

#### 2.2.3. FTIR Spectroscopy

**Figure 5:**
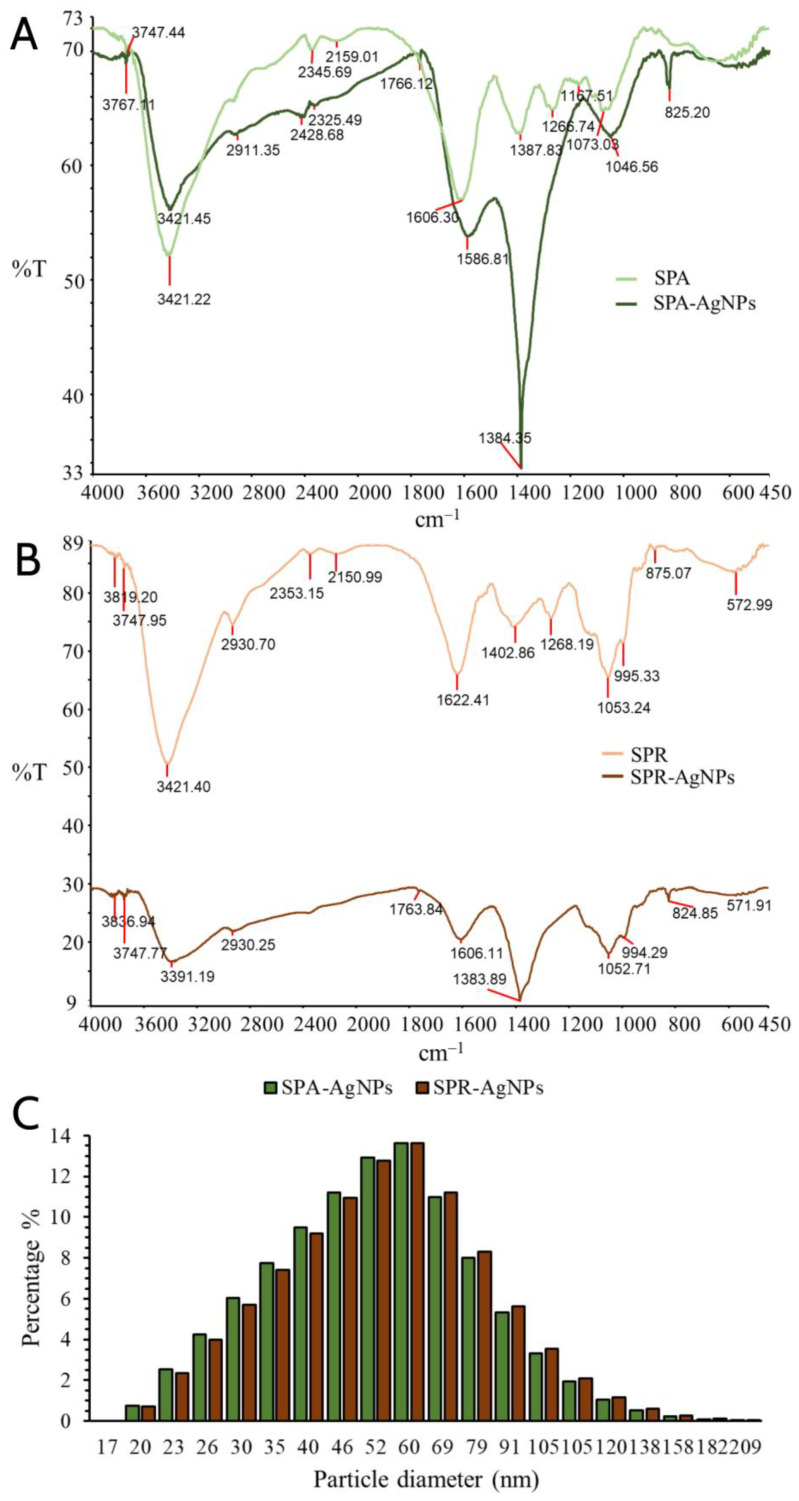
FTIR spectra of the *S. pratensis* aerial part (**A**) and root (**B**) extracts and corresponding AgNPs. (**C**) Size distribution of SPA-AgNPs and SPR-AgNPs by DLS analysis.

The responsibility of the biomolecules present in the extracts for the reduction of silver ions and stabilization of the synthesized nanoparticles was confirmed by FTIR analysis (Figure 5). The presence of identical vibrational bands, as a consequence of the corresponding vibrations of the functional groups of phytochemicals, in the spectra of the nanoparticle samples and extracts was observed. Functional groups were identified by comparing the detected bands with standard values.

The most distinctive functional groups were found by comparing the obtained values of the absorption bands with standard values and the literature data. As can be seen in Figure 5A (SPA and SPA-AgNPs), there was a coincidence of absorption bands at 3391.19 cm^−1^ for SPA-AgNPs and 3421.45 cm^−1^ for SPA. This band is responsible for the vibration of the –OH group present in the phenolic compounds. The aliphatic –C–H stretching is represented by a tiny band at 2930.70 cm^−1^ in the SPA spectra, which also shows up in the SPA-AgNP spectrum at 2930.25 cm^−1^. Pronounced bands at 1622.41 cm^−1^ for SPA and 1606.30 cm^−1^ for SPR were also present in the spectra of the corresponding nanoparticles SPA-AgNPs and SPR-AgNPs. These broad bands are responsible for the –C=C-stretching in the aromatic rings but appear to be integrated with the bands originating from the stretching vibration band at around 1730 cm^−1^, characteristic for the C=O group of carboxylic acid derivatives as well as for the C=O group of the flavones’ C ring. The bands at 1387 and 1402 cm^−1^ in the SPA and SPR spectra, respectively, may originate from the C–O bending vibrations of the carboxylic acids or esters, and the O–H bending vibration of alcohol and phenols [15,26,27]. The presence of the same bands in SPA-AgNPs (1384 cm^−1^) and SPR-AgNPs (1383 cm^−1^) suggests that compounds with these functional groups have a high affinity to binding to the synthesized AgNPs and stabilizing them.

#### 2.2.4. DLS Analysis

Due to the tendency of silver nanoparticles synthesized in this way to agglomerate during drying, their size was determined by dynamic light scattering (DLS) analysis. This technique is based on monitoring the Brownian movement of nanoparticles in the solution, which occurs randomly in all directions, with smaller particles moving faster than larger ones. The range of dynamic light scattering measurements is therefore limited to particles 10 μm in size because larger particles are too heavy to exhibit Brownian motion [19,28].

Based on the obtained results, it can be concluded that the sizes of the nanoparticles synthesized using SPA and SPR extracts ranged from 20 to 209 nm (Figure 5C). The highest percentage of SPA-AgNPs and SPR-AgNPs had diameters from 35 to 79 nm. The diameters of the nanoparticles less than 79 nm were observed in 98% of SPA-AgNPs, while a slightly lower percentage (86.22%) of SPR-AgNPs had diameters less than 79 nm. It was considered that physical parameters such as pH and temperature had the greatest influence on the size of nanoparticles, but also the other parameters such as compounds from plants that participate in the reduction of silver ions and the stabilization and morphology of the resulting nanoparticles may have a significant influence [19]. It was noted that many other researchers have synthesized silver nanoparticles of similar dimensions using different plants [29]. The size of the nanoparticles is largely responsible for their biological properties such as antimicrobial and anticarcinogenic properties as well as interaction with some molecules (proteins and DNA). As the size of the nanoparticles increases, their ability to interact with different biomolecules decreases, along with their biological activity. Siakavella et al. [30] reported that they obtained the smallest silver nanoparticles using the sage extract, while the largest ones were formed using the *Calendula* extract.

### 2.3. Phenolic Profile of S. pratensis Extracts and Antioxidant Activity of Extracts and Synthesized AgNPs

Table 1 shows the results of the spectrophotometric determination of the total content of phenolic and flavonoid compounds in aqueous extracts of *S. pratensis* aerial part and root. The content of the total phenolic compounds was significantly higher in the extract of the aerial part than in the root extract. Moreover, the flavonoids have not been identified in SPR, while it was 8.65 mg of quercetin equivalents (QUE) per g of dry plant in the SPA extract.

In the research reported by Sreckovic et al. [17], it was confirmed that the methanolic extracts of *S. pratensis* are rich in a variety of phenolic compounds, whereas over 60 were identified using UHPLC–MS^4^ Orbitrap metabolic fingerprinting. The results showed that the root extract was richer in the phenolic compounds, which is in contrast to our results of the aqueous extracts. However, the methanolic extract of the aerial part contained a higher amount of flavonoids, while the root extract had a significantly higher content of phenolic acids and condensed tannins. In addition, some biologically active compounds such as rosmarinic acid, salvianolic acids A and B, caffeic acid, luteolin, apigenin, rutin, quercetin 3-O-glucoside, and others were quantified in *S. pratensis*. Rosmarinic acid has been successfully used for the capping and stabilization of silver and gold nanoparticles [31,32]. However, rosmarinic acid is not the only compound used for nanoparticle synthesis, other identified compounds in *S. pratensis* such as quercetin, rutin, and caffeic acid have been used for the synthesis of silver nanoparticles with good antioxidant, anti-inflammatory, antimicrobial, and anti-cancer activities [33,34,35].

The differences in the obtained results for the antioxidant activity of nanoparticles between these two used methods for determination of antioxidant activity are most likely reflected in the different reaction mechanisms. Both types of nanoparticles exhibited significantly higher antioxidant activity against the ABTS radical cation than against the DPPH radical. It was noticed that the SPA extract produced silver nanoparticles (SPA-AgNPs) with a higher antioxidant potential (IC_50_ for DPPH˙ 83.54 µg/mL; IC_50_ for ABTS^•+^ 21.88 µg/mL) compared with SPR-AgNPs, and this can be explained by the higher content of polyphenols in SPA and consequently the higher polyphenolic content on the surface of the formed SPA-AgNPs (Table 2). Additionally, since the nanoparticles’ biological and physical qualities improve with a size reduction due to an increase in the contact surface, the smaller diameter of SPA-AgNPs also contributed to their greater antioxidant properties. The root extract showed weaker antioxidant properties, and therefore the nanoparticles synthesized using this extract (SPR-AgNPs) had a weaker antioxidant capacity compared with SPA-AgNPs. SPR-AgNPs neutralize the DPPH radical by 50% at 93.11 µg/mL, while the IC_50_ value for the ABTS^•+^ was 74.55 µg/mL. Gecer et al. [15] examined the antioxidant potential of silver nanoparticles synthesized using *S. aethiopis* and confirmed that the synthesized nanoparticles had higher potential to neutralize DPPH radicals compared to the corresponding extract. In this research, the nanoparticles showed higher activity, in ABTS^•+^ method, in comparison with BHT and Trolox, which were used as the standard compounds.

Some research demonstrated that the synthesized nanoparticles had a higher antioxidant potential than the extract used for their synthesis, but there is some research while with contrary results. Khorrami et al. [36] successfully synthesized silver nanoparticles using the *Juglans regia* extract and concluded that the antioxidant potential of nanoparticles could be reflected as a simultaneous effect of their catalytic effects and polyphenols, which are responsible for their antioxidant activity. According to Elemike et al. [37], silver nanoparticles have antioxidant capabilities mainly due to the presence of phenolic compounds, flavonoids, and terpenoids from plants on their surface, which can act as hydrogen donors, reducing agents, and singlet oxygen quenchers. Otunola et al. [38] dealt with the comparison of the antioxidant activity of nanoparticles synthesized using garlic (*Allium sativum*), ginger (*Zingiber officinale*), and cayenne pepper (*Capsicum frutescens*) extracts. The highest antioxidant potential according to ABTS^•+^ was observed in nanoparticles synthesized using the cayenne pepper extract, while nanoparticles synthesized using garlic showed a low IC_50_ value against the DPPH radicals. This suggests that the antioxidant activity largely depends on the type of phytochemical that is incorporated into the nanoparticle structure.

### 2.4. Antimicrobial Potential of Synthesized Silver Nanoparticles

The use of silver in various forms as an antimicrobial agent has a long tradition. Therefore, in recent times, there have been many attempts to produce new materials based on silver in order to improve its antimicrobial activity and possibly expand its application to different fields. Silver nanoparticles have exceptional antibacterial activity, even at low concentrations, due to a high surface-to-volume ratio [9]. The mechanism of the antimicrobial effect of silver nanoparticles is not fully understood. The most accepted theory is based on the gradual release of silver ions that, thanks to their electrostatic attraction and affinity for sulfur, bind to proteins containing the amino acids cysteine and methionine. In this way, they bind to the cytoplasmic membrane, causing a disruption of the permeability of the membrane and disruption of the bacterial envelope [36]. In addition, if the nanoparticles pass that barrier and reach the cell, free silver ions can deactivate enzymes and modify the DNA molecule, creating reactive oxygen species. Moreover, silver ions can interact with ribosomes in the cytoplasm and thus inhibit protein synthesis [9]. Table 3 shows the minimal inhibitory concentrations (MIC) of the synthesized nanoparticles in comparison with the values obtained for the antibiotic (ciprofloxacin) or antimycotic (clotrimazole).

The antibacterial activity of the synthesized nanoparticles was quite different, and the SPA-AgNPs showed a weaker activity against most of the tested bacteria in comparison with the SPR-AgNPs. The highest minimal inhibitory concentration of SPA-AgNPs was observed in *S. epidermidis* and *K. pneumonaiae* (20 mg/mL), while the most sensitive bacteria were *E. faecalis*, *S. enteritidis*, and *P. aeruginosa*, in which the MIC values were lower than the lowest applied (<0.0391 mg/mL). On the other hand, SPR-AgNPs showed a minimum inhibitory concentration lower than the lowest applied (<0.0391 mg/mL) in almost all bacteria. The most resistant bacterial species to this type of nanoparticles was *E. coli* (2.5 mg/mL). The obtained results are in accordance with the results obtained by Srećković et al. [17], where the methanol extract of the *S. pratensis* roots showed strong antibacterial activity against almost all of the bacteria used. This suggests the possibility that the compound from the root, which is responsible for the antibacterial activity, was incorporated into the nanoparticle structure. It is interesting that both types of nanoparticles showed high antibacterial activity against extremely resistant *P. aeruginosa* (<0.0391 mg/mL) as well as against *E. faecalis* and *S. enteritidis* (<0.0391 mg/mL).

In the results obtained for an antifungal activity for both types of nanoparticles, slightly weaker activity was observed compared with their antibacterial activity. However, in this case, SPR-AgNPs showed better activity compared with SPA-AgNPs. The most sensitive fungi to SPA-AgNPs were *T. longibrachiatum* and *T. harzianum* (MIC; 1.25 and 0.3125 mg/mL; respectively), while the fungi *P. canescens* and *P. cyclopium* were the most sensitive to SPR-AgNPs (MIC; <0.0391 mg/mL). For all other fungi, the MIC was above 10 mg/mL, while for *C. albicans*, the MIC for both types of nanoparticles was 20 mg/mL.

### 2.5. Biocompatibility of SPA-AgNPs and SPR-AgNPs

**Figure 6:**
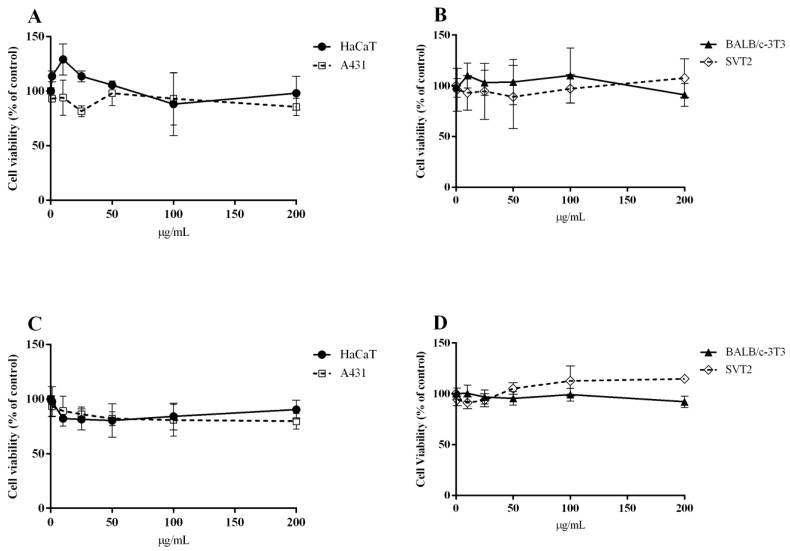
Effect of SPA-AgNPs and SPR-AgNPs on the viability of immortalized cells and cancer cells. Increasing concentrations (1–200 μg/mL) of SPA-AgNP (**A**,**B**) or SPR-AgNP (**C**,**D**) nanoparticles were added to HaCaT (black circles), BALB/c-3T3 (black triangles), A431 (empty squares), or SVT2 (empty rhombuses) for 72 h. Cell viability was assessed by the MTT assay and cell survival was expressed as a percentage of viable cells in the presence of the nanoparticle under test with respect to the control cells grown in the absence of the nanoparticle. The data shown represent the means ± S.D. of three independent experiments.

For biomedical use, especially for in vivo applications, the biocompatibility of nanoparticles is a crucial factor that determines the possibility of safe application. Thus, the biocompatibility of both nanoparticles on immortalized cells was analyzed. Based on the results shown in Figure 6, both nanoparticles were fully biocompatible with the immortalized cells, as no toxicity was observed up to 200 µg/mL after 72 h of incubation. Additionally, SPA-AgNPs and SPR-AgNPs were not toxic against the A431 and SVT2 cancer cells after 72 h treatment with concentrations up to 200 µg/mL (Figure 6). In general, the silver nanoparticles synthesized by standard methods consist only of silver atoms with non-selective cytotoxicity. The use of polyphenolic compounds from *Salvia*, which were proven to show low toxicity toward a range of cell lines [17,39,40], did not affect the overall biocompatibility.

### 2.6. Hemolytic Activity of SPA-AgNPs and SPR-AgNPs

The ability of nanoparticles or some other molecules to decompose the red blood cell membrane, made up of proteins, lipids and glycoproteins, and thereafter release the hemoglobin, is named hemolysis. In addition to the fact that nanoparticles are already used in several medical conditions such as cancer and HIV, they can also be used as transport systems for drug delivery. Considering that at some point there will be an interaction between nanoparticles and red blood cells, the importance of assessing hemocompatibility is of great importance. The potential hemolytic activity of nanoparticles greatly limits their medical use [41]. In order to improve the hemocompatibility of nanoparticles, surface modification is one of the most widely used strategies. One of the ways of changing the surface of nanoparticles is the binding of the polyphenolic compounds of plants to their surface, which are significantly more compatible in comparison with the pure metal or metal-oxide nanoparticles. In addition, these compounds can have a number of other benefits for the cardiovascular system thanks to their antioxidant properties [42]. The process of the hemolysis of human erythrocytes during interaction with SPA-AgNPs and SPR-AgNPs was recorded spectrophotometrically at 540 nm after incubation for 1 h at 37 °C. The results show that SPA-AgNPs and SPR-AgNPs did not cause the hemolysis of erythrocytes at concentrations up to 150 µg/mL. The level of released hemoglobin from erythrocytes incubated with both synthesized nanoparticles was lower or similar to the hemoglobin level in the negative control samples (erythrocytes + phosphate buffered saline (PBS)). Moreover, it can be concluded that the nanoparticles even had a stabilizing effect on the cell membrane due to less hemolytic activity compared with the negative control. A 1% SDS solution was used as a positive control, considering that it causes complete hemolysis of the erythrocytes (100%). According to the American Society for Clinical Pathology, the accepted percentage of hemolysis rate for a drug is 2% or less [43].

### 2.7. Catalytic Application of Nanoparticles

**Figure 7:**
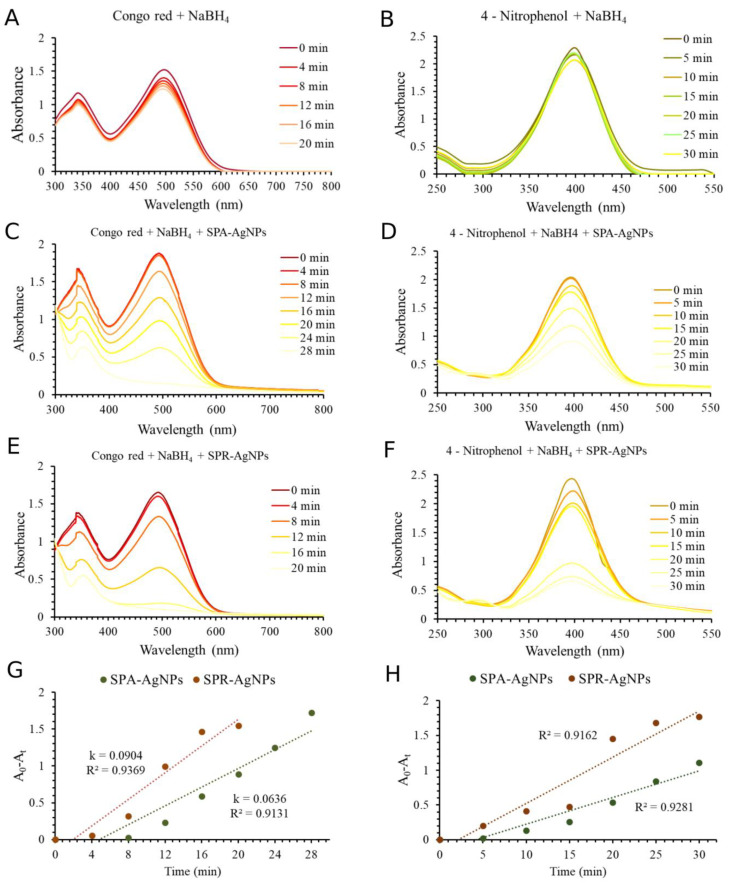
UV–Vis spectra of the catalytic degradation of Congo Red (**A**) and 4-nitrophenol (**B**) in the presence of NaBH_4_ without AgNPs. Catalytic degradation of Congo Red in the presence of SPA-AgNPs (**C**) and SPR-AgNPs (**E**). Catalytic degradation of 4-nitrophenol in the presence of SPA-AgNPs (**D**) and SPR-AgNPs (**F**). The best fitted chemical kinetic linear model (zero-order) for Congo Red (**G**) and 4-nitrophenol (**H**) degradation using synthetized AgNPs and NaBH_4_.

The use of nanoparticles as catalysts has been on the rise in recent years, and nanocatalysts have found applications in water purification, biodiesel production, catalysts in fuel cells, organic syntheses, and catalysts for the degradation of hazardous waste [44]. One of the anthropogenic activities that pollutes and consumes the most water is the textile industry. The biochemical and chemical oxygen consumption, impairment of photosynthesis, inhibition of plant growth, entry into the food chain, bioaccumulation, and potential for toxicity and carcinogenicity are all considerably compromised by textile dyes. Synthetic dyes, especially those classified as reactive, show high solubility in water, which makes it difficult to eliminate them with ordinary methods. The significant surface atom fractions and interfacial free energy may affect the thermostability and catalytic capabilities of nanoparticles [45]. The use of catalysts that have the possibility of initiating the degradation of synthetic dyes and help in their biodegradation by microorganisms is of great importance [46]. The success of the application of silver nanoparticles synthesized by plants in the degradation of Congo Red (CR) and 4-nitrophenol (4-NP) has been confirmed many times [2,47,48]. For this reason, the catalytic potential of both types of synthesized nanoparticles in the degradation of Congo Red and 4-nitrophenol (Figure 7) was investigated.

The results showed that the degradation of CR and 4-NP in the presence of NaBH_4_ as a reducing agent was very slow. As shown in Figure 7A,B, the absorbances of CR and 4-NP, respectively, without the addition of AgNPs as a catalyst in the presence of NaBH_4_, were almost unchanged during 20–30 min. The addition of SPA-AgNPs and SPR-AgNPs in these reactions induced rapid change in the decrease of absorbances in the UV–Vis spectra of CR and 4-NP (Figure 7C–F). Using SPA-AgNPs and SPR-AgNPs in the reaction, the color of CR and 4-NP changed to colorless within 30 min, approving the catalytic potential of synthesized AgNPs. In this reaction, NaBH_4_ dissociate to BH_4_^−^ and may act as an electron donor while the AgNPs have the role of transferring these electrons to CR and 4-NP, enabling their reduction degradation [49,50]. The experimental data were obtained from the absorbances (A) for CR and 4-NP at different times under constant temperature (25 °C), the NaBH_4_ and AgNPs concentrations were fitted into three kinetic models: zero-order, first-order, and second-order [50,51], and the results of the apparent rate constant (k) and correlation coefficient (R^2^) of SPA-AgNPs and SPR-AgNPs for the catalytic reduction of CR and 4-NP are shown in Table 4. In this catalytic reaction, the rate of reaction can be considered to be independent of the NaBH_4_ concentration because the concentration of NaBH_4_ was significantly higher than CR and 4-NP, hence, the degradation pathway may be described as the pseudo-order reactions. The comparison of the calculated R^2^ values (Table 4) for each model showed that the zero-order best describes the mechanism and rate of the catalytic degradation of CR and 4-NP (Figure 7G,H, respectively) using synthesized AgNPs. The process of catalytic degradation can usually be described with the pseudo-first-order kinetics model. However, there are some reports that describe catalytic processes using nanoparticles can also be described with a pseudo-zero-order model [50,52,53]. As shown in Table 4 and Supplementary Figure S1, the regression coefficients (R^2^) of first-order and second-order kinetics were lower than the pseudo-zero-order kinetic, which is in accordance with some previously published results about the use of different metal nanoparticles in the catalytic degradation of some dyes [50,52,53]. 

Figure 7C,E shows the decrease in the absorption maximum of Congo Red at 496 nm depending on time, which could also be observed visually by the disappearance of the bright red color. The obtained results indicate that for the complete degradation of Congo Red by SPR-AgNPs (20 min) (Figure 7E) less time was required compared with SPA-AgNPs (28 min) (Figure 7C). This is evident from Figure 7G and Table 4 which show the calculated catalytic reaction rate constants. The catalytic potential of SPA-AgNPs and SPR-AgNPs during the conversion of 4-nitrophenol to 4-aminophenol are shown in Figure 7D,F, respectively, along with the calculation of the rate constants (Figure 7H and Table 4). This reaction is often presented as the gold standard in the investigation of the catalytic potential of nanoparticles of different metals [54]. According to the reaction rate constants (Figure 7H), it can also be shown that in this instance, the SPR-AgNPs had a slightly higher efficiency in the reduction of 4-nitrophenol compared with SPA-AgNPs. In contrast to the reaction involving SPA-AgNPs in which the absorbance of 4-nitrophenol decreased proportionally over time, in the reaction involving SPR-AgNPs, the absorbance decreased slightly up to 15 min before dropping sharply up to 20 min and then continuing to decrease at a slower pace.

## 3. Materials and Methods

### 3.1. Extraction Process of S. pratensis

*S. pratensis* aerial parts and roots were gathered in May 2018 at Veliko Krčmare, a village located in central Serbia. The plant was identified by Prof. Dr. Milan Stanković at the Herbarium of the Department of Biology and Ecology, Faculty of Science, University of Kragujevac, Serbia (voucher specimen No. 129/018). The plant material was cleaned of any contaminants by rinsing it with deionized water, after which it was dried at room temperature in a dark place and milled into powder. Separately, 10 g of previously dried and powdered aerial part (SPA) and root (SPR) was added to 100 mL of boiling deionized water. The extracted materials were filtered through Macherey-Nagel 85/70 mm filter paper after one hour, whereby the obtained extracts were stored in a refrigerator at 4 °C for further use up to one week.

### 3.2. Total Content of Phenolic and Flavonoid Compounds in the Extracts

The spectrophotometric methods described by Srećković et al. [55] were used to determine the total content of phenolic compounds and flavonoids. Folin–Ciocalteu reagent was used to determine the content of the total phenolic compounds. The extracts were diluted to the appropriate concentration, and aliquots of 0.25 mL were mixed with 1.25 mL Folin–Ciocalteu reagent (previously diluted ten times with distilled water) and 7.5% NaHCO_3_. By incubating this mixture for 15 min at 45 °C, the absorbance was measured at 765 nm. The result was expressed in gallic acid equivalents (mg GAE/g of dry plant material).

The flavonoid content was determined by mixing 1 mL of the extract of the degumming dilution with the same volume of 2% AlCl_3_ dissolved in methanol. After staying at room temperature and in a dark place for a time interval of 1 h, the absorbance was read at λmax 415 nm. The total content of flavonoids in the extract was expressed in quercetin equivalents (mg QUE/g of dry plant material). Results are presented as the mean value of three independent measurements.

### 3.3. Synthesis of Silver Nanoparticles and Optimization Conditions

The optimal synthesis conditions, in which the highest yield with the smallest nanoparticles is formed, were sought by changing parameters such as temperature (25, 50, and 80 °C), pH value (2, 7, 12), AgNO_3_ concentration (5, 10, and 20 mM), and extract concentration (5, 10, and 20%). First, the synthesis was carried out in a neutral pH environment at room temperature with a 10 mM salt concentration and a 10% extract concentration. Then, changing one parameter at a time, the influence of the change in synthesis conditions was monitored, and this change was recorded by monitoring the absorption spectrum from 800 to 300 nm. When the ideal conditions were identified, a larger quantity of nanoparticles was created and subsequently employed for the characterization and biological and catalytic tests.

### 3.4. Characterization

UV–Vis absorption spectra (800–300 nm) were recorded using a Halo DB-20S (Dynamica GmbH, Switzerland) spectrophotometer using a quartz cuvette. The crystal structure of the synthesized nanoparticles was determined using an X-ray diffractometer. The instrument (PHILIPS PW 1710) was set to a voltage of 40 kV and a current of 30 mA, with CuKα radiation of 1.54178 Å. The nanoparticles were examined in the 10–90° 2θ range with a 0.02° step and a 0.25 s retention period at each step. The surface, morphology, and elemental compositions of the synthesized nanoparticles were recorded using a scanning electron microscope (SEM) JOEL JSM IT 300LV with an EDS detector (OXFORD Instruments, X-max). The Mastersizer 2000 (Malvern Panalytical, Malvern) was used to determine the size of the nanoparticles dispersed in water based on dynamic light scattering (DLS) analysis. In order to confirm the assumption that phenolic compounds are responsible for the formation and stabilization of nanoparticles, the FTIR spectra of nanoparticles and dry water extracts were recorded by the KBr pellet method. The samples were mixed with potassium bromide (KBr) powder, which was then pressed into a disk.

### 3.5. Antioxidant Activity

The antioxidant activity of the nanoparticles and extracts was determined using two commonly known assays, DPPH and ABTS [17]. The DPPH assay is based on the neutralization of the purple DPPH radical (2,2-Diphenyl-1-picrylhydrazyl) by the tested sample to the yellow-colored form of diphenylpicrylhydrazine, which leads to a decrease in absorbance at 517 nm. First, a series of double dilutions of the samples and standard with a volume of 1 mL was prepared, in which 1 mL of DPPH solution (dissolved in methanol 80 µg/mL) was added and left in the dark for 30 min. After the incubation period, the absorbance was measured at 517 nm. With regard to the ABTS radical cation, different sample concentrations were mixed with the same volume of ABTS radical cations (2,2’-azino-bis(3-ethylbenzothiazoline-6-sulfonic acid)), and after 30 min of incubation in the dark at room temperature, the absorbance was measured at 734 nm. The results for both methods were expressed as the IC_50_ value obtained using the dose-response sigmoidal curve produced with OriginPro8 software (OriginLab, Northampton, MA, USA).

### 3.6. Antimicrobial Activity

The antimicrobial activity of the synthesized nanoparticles was tested using the microdilution method against eleven bacteria species, eight species of fungi, and one mold. The bacteria for this experiment were provided by the Institute of Public Health Kragujevac, while the fungi were obtained from the Department of Biology and Ecology, Faculty of Science, University of Kragujevac, Serbia. Freshly cultivated microorganisms were necessary for the microdilution assay. Bacterial species were cultivated on nutrient agar for 24 h at 37 °C, fungi on glucose-potato agar for 3 to 7 days at 28 °C, and *C. albicans* on Sabouraud dextrose broth for 24 h at 35 °C.

Antimicrobial properties were assessed using the microdilution assay [56]. The first step was to prepare a series of double dilutions of the sample or antimicrobial standards in a suitable nutrient broth (Miller-Hilton broth for determining antibacterial activity or Sabourand dextrose broth for determining antifungal activity) using 96-well microtiter plates. Afterward, a bacterial spore suspension (1 × 10^6^ CFU/mL) prepared using the McFarland standard was added, while the density of the fungal spore suspension was (1 × 10^4^ CFU/mL) [57,58]. Resazurin was used to identify bacterial growth, whereas visual observation was utilized to detect fungal growth. Finally, the microtiter plates were incubated for 24 h at 37 °C before the results were read. The microtiter plates were incubated at 28 °C for 72 h to read the antifungal activity. The lowest concentration of the sample/standard at which there was no change in the color of resazurin (i.e., visual growth of fungi) was designated as the minimum inhibitory concentration value. A more detailed procedure was previously described in a study by Sreckovic et al. [55].

### 3.7. Hemolytic Activity

The potential hemolytic activity of the synthesized nanoparticles was determined by monitoring the release of hemoglobin from the erythrocyte cells during their incubation with nanoparticles under physiological conditions. Erythrocytes were isolated from human blood samples obtained from healthy volunteers. Blood samples were collected in tubes with ethylenediaminetetraacetic acid (anticoagulant) and then centrifuged at 2500 rpm for 10 min to isolate red blood cells. Then, the precipitate (red blood cells) was washed several times with phosphate buffer solution (PBS, pH 7.4) and centrifuged with the same parameters. Different concentrations of the SPA-AgNPs and SPR-AgNPs (150, 120, 90, 60, 30, and 10 mg/mL) were dissolved in PBS and mixed with the same volume of 5% red blood cell solution, also prepared in PBS. A 1% solution of sodium dodecyl sulfate (SDS) was used as a positive control, while only PBS was used instead of the samples as a negative control. After an incubation period of 1 h at 37 °C, the samples were centrifuged for 15 min at a speed of 1200 rpm. Then, the absorbance of the supernatant was measured at 540 nm [2]. The results were obtained using the following equation:

$$\%Hemolysis=\frac{\left(A_{AgNPs}-A_{0}\right)-A_{k}}{A_{SDS}-A_{k}}\times 100$$

where *A_AgNPs_* is the absorbance of the erythrocyte sample incubated with AgNPs; A_0_ is the absorbance of erythrocyte samples incubated with PBS; *A_k_* is the absorbance of the corresponding AgNP solution diluted in PBS for the correction of absorption, which originates from AgNPs samples; and *A_SDS_* is the absorbance of erythrocyte samples incubated with *SDS*.

### 3.8. Biocompatibility and Cytotoxicity Analyses

Human keratinocytes (HaCaT, Biscay, Spain) and murine fibroblast BALB/c-3T3 cells (ATCC, Manassas, VA, USA) were used as immortalized cell lines. The potential cytotoxic effects of synthesized nanoparticles on cancer cells were determined using human A431 (epidermoid carcinoma) and BALB/c 3T3 transformed with SV40 virus (SVT2) cells (ATCC, Manassas, VA, USA). Cells were cultured in 10% fetal bovine serum in Dulbecco’s modified Eagle’s medium, in the presence of 1% antibiotics and 2 mM L-glutamine, in a 5% CO_2_ humidified atmosphere at 37 °C. Cells were seeded in 96-well plates at a density of 2.5 × 10^3^ cells/well for HaCaT cells, 3 × 10^3^ cells/well for BALB/c 3T3 cells, and 1.5 × 10^3^ for both cancer cell lines. For the biocompatibility assay, 24 h after seeding, increasing concentrations of silver nanoparticles (from 1 to 200 µg/mL) were added to the cells. After 72 h of incubation, the cell viability was assessed by the MTT assay, as described in Galano et al. [59]. Briefly, 3-(4,5-dimethylthiazol-2-yl)-2,5-diphenyltetrazolium bromide (MTT) reagent, dissolved in Dulbecco’s Modified Eagle medium (DMEM) in the absence of phenol red (Sigma-Aldrich), was added to the cells (0.5 mg/mL final concentration). Following 4 h of incubation at 37 °C, the culture medium was removed, and the resulting formazan salts were dissolved by adding isopropanol containing 0.01 N HCl. Absorbance values were determined at 570 nm using an automatic plate reader (Microbeta Wallac 1420, PerkinElmer, Waltham, MA, USA). Cell survival was expressed as a percentage of viable cells in the presence of the silver nanoparticle under test, compared with the control cells grown in the absence of silver nanoparticles. Control experiments were performed either by growing cells in the absence of the silver nanoparticles or by adding identical volumes of buffer to the cell culture. Each experiment was done at least three times.

### 3.9. Catalytic Application of Nanoparticles during Congo Red and 4-Nitrophenol Degradation

The present study examined the potential application of synthesized silver nanoparticles in the degradation of Congo Red and 4-nitrophenol using the assays described by Umamaheswari et al. [60] and Desai et al. [61]. The reaction of 1 mL SPA-AgNPs or SPR-AgNPs (0.1 mg/mL), 1.5 mL NaBH_4_ (5.28 × 10^−2^ M), and 5 mL of Congo Red (9.37 × 10^−5^ M) was monitored by measuring the change in the absorbance of the reaction mixture in the range of 800 to 300 nm every four minutes during 20 min using a UV–Vis spectrophotometer.

In order to evaluate the catalytic potential of SPA-AgNPs and SPR-AgNPs in 4-nitrophenol degradation, 0.5 mL of nanoparticles (0.3 mg/mL) was mixed with 0.5 mL NaBH_4_ (1.32 × 10^−3^ M) and 5 mL 4-nitrophenol (7.19 × 10^−5^ M). The reaction was monitored using a UV–Vis spectrophotometer by recording the spectrum from 550 to 250 nm at room temperature for 20 min. The reactions were also monitored without the presence of nanoparticles. The kinetics of the degradation reactions of CR and 4-NP were fitted into three kinetic models: zero-order, first-order, and second-order [50,51].

## 4. Conclusions

Using the principles of green chemistry, silver nanoparticles were successfully synthesized in this work, using aqueous extracts of the aerial part and roots of *S. pratensis* as inexpensive, biologically renewable reducing agents. The nanoparticles exhibited higher antioxidant activity than the corresponding plant extracts, whereby SPA-AgNPs showed significantly higher activity than SPR-AgNPs. However, SPR-AgNPs possessed a higher antimicrobial activity than SPA-AgNPs against almost all microorganisms. Their biocompatibility with healthy cells and non-hemolytic properties makes them candidates for potential use as antimicrobial agents in the treatment of various medical conditions. The possibility of SPA-AgNPs and SPR-AgNPs being used as catalysts was confirmed by Congo Red and 4-nitrophenol reduction reactions. In both reactions, SPR-AgNPs proved to be better catalyst, which was also confirmed by the rate constants of the reaction.

## Figures and Tables

**Table 1:** Spectrophotometric determination of the total phenolic and flavonoid contents in the *S. pratensis* aerial part (SPA) and root (SPR) extracts.

Extracts	Total Phenolic Content (mg GAE/g of Dry Plant)	Total Flavonoid Content (mg QUE/g of Dry Plant)
SPA	79.39 ± 3.30	8.65 ± 0.94
SPR	21.61 ± 0.51	n.d.

n.d.—not detected; GAE—gallic acid equivalents; QUE—quercetin equivalents.

**Table 2:** Comparison of the antioxidant activity of the synthesized silver nanoparticles against the corresponding *S. pratensis* extracts.

Extracts	IC_50_ Values (µg/mL)	
	DPPH˙ Scavenging Activity	ABTS^•+^ Scavenging Activity
SPA	101.18 ± 7.00	42.62 ± 7.89
SPA-AgNPs	83.54 ± 4.23	21.88 ± 3.33
SPR	130.19 ± 14.84	192.44 ± 12.55
SPR-AgNPs	93.11	74.55 ± 9.25

**Table 3:** Antimicrobial activity of the synthetized AgNPs.

Bacterial Species			MIC (mg/mL)		MIC (µg/mL)	
			SPA-AgNPs	SPR-AgNPs	Ciprofloxacin	Clotrimazole
*S. epidermidis*	G+	ATCC 12228	0.625	0.156	2.5	-
*S. aureus*	G+	ATCC 25923	0.625	<0.0391	2.5	-
*B. subtilis*	G+	ATCC 6633	2.5	<0.0391	10	-
*B. cereus*	G+	ATCC 10876	1.25	<0.0391	20	-
*M. lysodeikticus*	G+	ATCC 4698	0.625	0.156	<0.3125	-
*E. faecalis*	G+	ATCC 29212	<0.0391	<0.0391	<0.3125	-
*S. enteritidis*	G−	ATCC 13076	<0.0391	<0.0391	<0.3125	-
*S. typhimurium*	G−	ATCC 14028	2.5	0.156	5	-
*E. coli*	G−	ATCC 25922	0.625	2.5	0.625	-
*P. aeruginosa*	G−	ATCC 10145	<0.0391	<0.0391	<0.3125	-
*K. pneumoniae*	G−	ATCC 70063	0.3125	<0.0391	<0.3125	-
Fungal species						
*T. longibrachiatum*		FSB 13	1.25	0.156	-	10
*T. harzianum*		FSB 12	0.3125	0.156	-	20
*P. canescens*		FSB 24	>10	<0.0391	-	2.5
*P. cyclopium*		FSB 23	>10	<0.0391	-	<0.0391
*D. stemonitis*		FSB 41	>10	0.625	-	0.625
*F. oxysporom*		FSB 91	>10	5	-	<0.0391
*A. brasiliensis*		ATCC 16404	>10	1.25	-	1.25
*A. alternata*		FSB 51	>10	0.3125	-	<0.0391
*C. albicans*		ATCC 10259	20	20	-	20

G+, gram-positive bacteria; G−, gram-negative bacteria.

**Table 4:** The rate constant and regression coefficient (R^2^) values of Congo Red and 4-nitrophenol degradation using different rate laws models.

Chemical Kinetics Model	Linear Equation ^a^	Nanoparticles	Congo Red		4-Nitrophenol	
			Regression Coefficient (R^2^)	Rate Constant (k) ^b^	Regression Coefficient (R^2^)	Rate Constant (k)^b^
Zero-order	A_0_ − A_t_ = kt	SPA-AgNPs	0.9131	0.0636	0.9281	0.0384
		SPR-AgNPs	0.9369	0.0904	0.9162	0.0665
First-order	$\ln\left(\frac{A_{0}}{A_{t}}\right)=\mathrm{kt}$	SPA-AgNPs	0.7235	0.0749	0.8836	0.0263
		SPR-AgNPs	0.8885	0.1514	0.8953	0.0490
Second-order	$\frac{1}{A_{t}}-\frac{1}{A_{0}}=\mathrm{kt}$	SPA-AgNPs	0.4812	0.1457	0.8244	0.0189
		SPR-AgNPs	0.7525	0.4436	0.8675	0.0407

^a^ A_0_—absorbance at the beginning of the reaction; A_t_—absorbance at different times (t) of the reaction; ^b^ k is expressed in mol L^−1^ min^−1^ for zero-order kinetics, min^−1^ for first-order kinetics, and L mol^−1^ min^−1^ for second-order kinetics.

## Data Availability

Not applicable.

## Supplementary Materials

The following supporting information can be downloaded at: https://www.mdpi.com/article/10.3390/molecules28031387/s1, Figure S1: Pseudo-first-order and pseudo-second-order reaction kinetics linear models for Congo Red (A,B) and 4-nitrophenol (C,D) degradation kinetics using synthesized silver nanoparticles (SPA-AgNPs and SPR-AgNPs) and NaBH_4_.

## Sample Availability

Samples of the compounds are not available from authors.
