# Supplementary material for: Biosynthesis of Silver Nanoparticles Using Salvia pratensis L. Aerial Part and Root Extracts: Bioactivity, Biocompatibility, and Catalytic Potential
Source: Molecules. 2023 Feb 1;28(3):1387. doi: 10.3390/molecules28031387 (PMC9921037; doi:10.3390/molecules28031387)
Supplement: Supplementary file 1 [file molecules-28-01387-s001.zip › molecules-2164600-supplementary.pdf]

Supplementary Material

Article

Biosynthesis of Silver Nanoparticles Using *Salvia pratensis* L. Aerial Part and Root Extracts: Bioactivity, Biocompatibility, and Catalytic Potential

[1] University of Kragujevac, Faculty of Science, Department of Chemistry, Radoja Domanovića 12, 34000 Kragujevac, Serbia
[2] University of Belgrade, Faculty of Physical Chemistry, Studentski Trg 12-16, 11159 Belgrade, Serbia
[3] University of Naples Federico II, Department of Chemical Sciences, Complesso Universitario Monte Sant'Angelo, via Cinthia 4, 80126 Naples, Italy
[4] eLoop S.r.l., V.le A, Gramsci 17/B, 80122 Napoli, Italy
[5] Mining and Metallurgy Institute Bor, Zeleni Bulevar 35, 19210 Bor, Serbia
[6] University of Kragujevac, Institute for Information Technologies Kragujevac, Department of Science, Jovana Cvijića bb, 34000 Kragujevac, Serbia
* Correspondence: nikola.sreckovic@pmf.kg.ac.rs (N.Z.S.); vladimir.mihailovic@pmf.kg.ac.rs (V.B.M.)

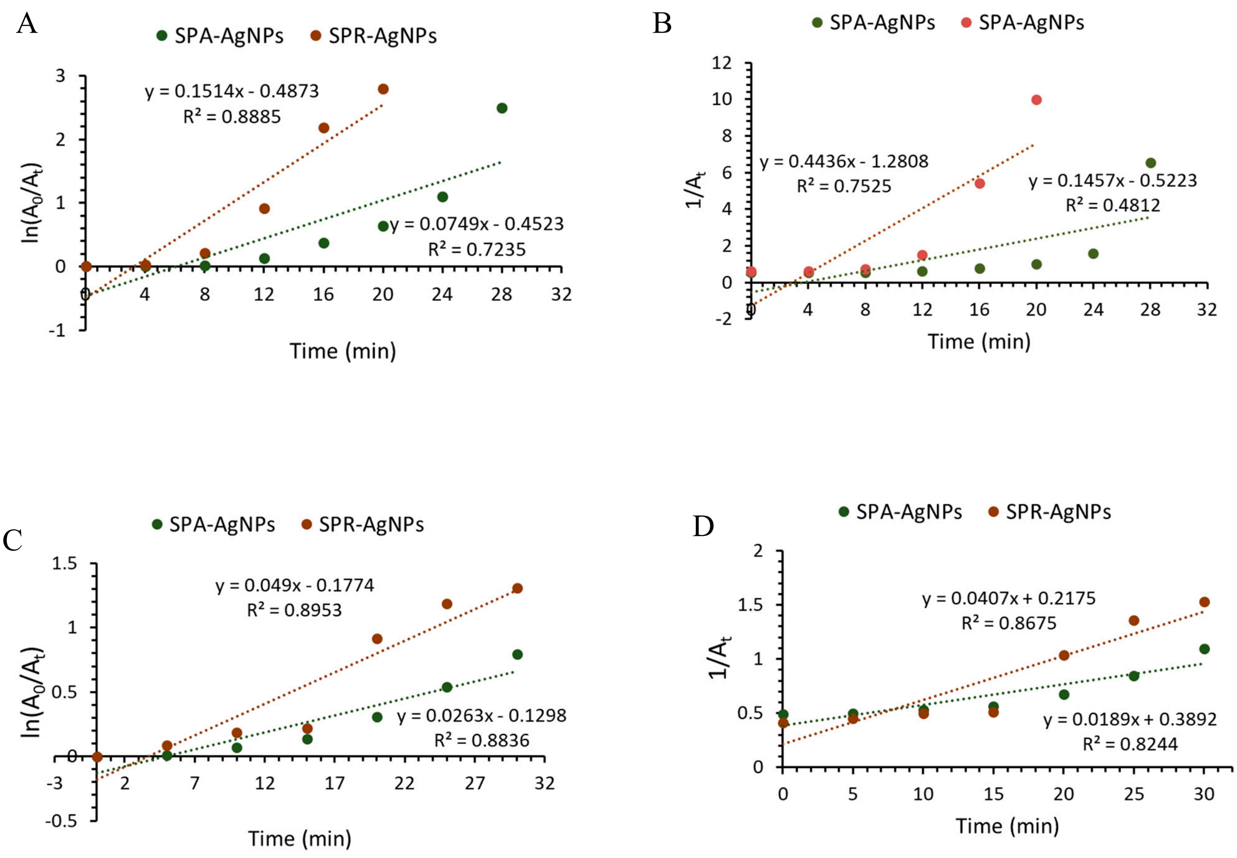

Figure S1. Pseudo-first-order and pseudo-second-order reaction kinetics linear models for Congo red (A and B) and 4-nitrophenol (C and D) degradation kinetic using synthesized silver nanoparticles (SPA-AgNPs and SPR-AgNPs) and $NaBH_4$
